# Application of a Simple In-House PCR-SSP Technique for HLA-B* 27 Typing in Spondyloarthritis Patients

**DOI:** 10.1155/2013/504109

**Published:** 2013-12-19

**Authors:** Devraj J. Parasannanavar, Anjali Rajadhyaksha, Kanjaksha Ghosh

**Affiliations:** ^1^National Institute of Nutrition, ICMR, Jamai Osmania, Tarnaka, Hyderabad, Andhra Pradesh, India; ^2^Department of Medicine, King Edward Memorial Hospital Campus, Parel, Mumbai 400012, India; ^3^National Institute of Immunohematology, 13th Floor, NMS Building, King Edward Memorial Hospital Campus, Parel, Mumbai, Maharashtra 400012, India

## Abstract

*Background*. Microlymphocytotoxicity (MLCT) and flowcytometry (FC) are the conventional serological methods to detect HLA-B* 27. Due to some disadvantages in these methods, most of the HLA laboratories have now switched over to molecular methods. Molecular techniques based on commercial kits are expensive; as such many laboratories with limited funds in developing countries cannot afford these techniques. *Aims*. Our main aim was to standardize a simple inexpensive in-house PCR-SSP technique for HLA-B* 27 typing. *Materials and Methods*. Sequence Specific primers were designed to amplify all the subtypes of B* 27 using IMGT-HLA sequence database. Accuracy was checked by retyping of 90 PCR-SSOP typed controls. *Results*. The presence of 149 bp specific band with control band on 2% agarose gel showed B* 27 positivity. No discrepancies were found when compared with PCR-SSOP results. The frequency of HLA-B* 27 was found to be significantly increased (68.75% versus 4.40%, O.R 46.909: *P* value 6.62*E* − 32) among 700 SpA patients as compared to controls. Clinically, 54% of patients had polyarticular arthritis with SI joints involvement (68%) and restricted spine flexion (60%). *Conclusion*. In-house PCR-SSP technique is very simple and inexpensive technique to detect B* 27 allele, which was strongly associated with SpA patients from Western India.

## 1. Introduction

Human leucocyte antigen-B27 is a major histocompatibility complex class I molecule that is strongly associated with AS and related seronegative spondyloarthritis (SpA). Ankylosing spondylitis (AS) is associated with B27 with a relative risk of 95 which is the highest among all HLA disease associations [1]. The association of HLA-B27 with AS was first reported in 1973 [2, 3] and confirmed with related SpA later by many other investigators [4–7]. The frequency of HLA-B27 ranges from 80% to 90% among patients as compared to 4–8% in controls. This high association stimulated considerable interest in HLA-B* 27 testing as a diagnostic tool in these inflammatory diseases.

The frequency of HLA-B27 in AS or other related SpA among Indian population varies from 40% to 80% as compared to 1.4–8% of controls as shown in Table 1 [8–15]. Techniques generally employed for the routine typing of HLA-B27 are the microlymphocytotoxicity test (MLCT) [16] and Flow cytometry (FC) [17]. Both these tests rely on the detection of cell surface antigens by antibodies. There are some disadvantages in MLCT like requirement of viable cells, cross-reactive nature of HLA antigens, unavailability of B* 27 specific antisera which cover all HLA-B* 27 alleles and the need for expert to give consistent cytotoxic results [18]. Flow cytometry requires more expensive reagents, more complex equipments, and better trained personnel. As a result, many laboratories have switched over to molecular methods.

Various methods like polymerase chain reaction based sequence specific priming (PCR-SSP), PCR based sequence specific oligonucleotide probing (PCR-SSOP), and microtiter well assay [19–21] can be used to detect HLA-B* 27 antigens. Several reports have suggested that the results obtained by PCR-SSP are far more accurate than the conventional serological approach [22]. This technique only relies on the detection of the HLA-B* 27 specific DNA sequences and is thus an ideal test for HLA-B* 27. As the commercially available SSP kits are extremely expensive, we have developed an in-house SSP typing technique for routine application for HLA-B* 27 genotyping. We have subsequently typed a large series of SpA patients and controls in our population.

## 2. Materials and Methods

In the present study, a total of 700 consecutive patients were selected according to the revised New York criteria for AS [23] and ESSG for SpA [24] over a period of three years (2009–2011). Modified New York criteria for AS require presence of radiological proven sacroiliitis at least grade 2 bilaterally or grade 3 or 4 unilaterally further clinically low back pain and morning stiffness for more than 3 months that improves with exercise but is not relieved by rest. Restricted spine flexion in both the sagittal and frontal planes and decreased chest expansion relative to normal values correlated for age and sex will also be required for diagnosis AS whereas ESSG criteria for SpA, includes presence of inflammatory back pain or asymmetric synovitis (predominantly in the lower limbs), plus any one of the following features: enthesopathy, alternating buttock pain, positive family history, psoriasis, inflammatory bowel disease, preceding infection in the urogenital or enteral tract. All patients were negative for rheumatoid factor. Clinical evaluation of the patients was done by rheumatologist and reevaluated by one of us. The study was approved by the Institutional Ethics Committee and the patients' consent was obtained. 90 age and sex matched healthy controls belonging to the same socioeconomic status and ethnic background during the same period comprised the controls.

Genomic DNA was extracted using standard phenol: chloroform method from the blood collected in K_2_ EDTA vacutainer. HLA-B* 27 specific primers were designed based on the sequence databases from IMGT/HLA (http://www.ebi.ac.uk/ipd/imgt/hla/probe.html): the sequences are as follows, *Forward 1*-5′- GCTACGTGGACGACACGCT-3′, *Reverse 1*-5′-CTCGGTCAGTCTGTGCCTT-3′, and *Reverse 2-5*′-TCTCGGTAAGTCTGT GCCTT-3′ corresponding to exon 2 of HLA-B. The control primers included HgH *Forward* -5′-TGCCTTCCCAACCATTCCCTTA-3′ and HgH *Reverse* 5′-CCACTCACGGATTTC TGTTGTGTTTC-3′ as described earlier [25]. These primers were able to amplify all the subtypes of HLA-B* 27, that is, ranging from B* 27:01 to 27:73 (73 alleles till July, 2010). One extra forward primer was added to amplify more alleles than that described earlier [26]. The final optimized reaction mix had the following composition: 10X complete buffer, 0.5 mM MgCl_2_, 0.08 mM dNTP, 0.05 pmol of each of the control as well as specific primers, and 4 units of *Taq* polymerase (BIORON, Germany) in 100 ul of premix. 10 *μ*L of this reaction mixture was equally divided into 10 different tubes of 10 ul each. Known B* 27 positive DNA sample was used as positive control. The effective concentration of DNA used in 100 *μ*L of cocktail was 10–100 ng/*μ*L. In case the DNA had a higher concentration than the specified range, it was appropriately diluted using distilled water. Amplification reactions were carried out in thermal cycler (Quanta Biotech, UK). The cycle parameters were as follows: Initial denaturation at 94°C for 5 minutes, 30 cycles at 94°C for 1 minute, at 65°C for 2 minutes, at 72°C for 1 minute, and a final extension at 72°C for 10 minutes. Amplified PCR product was kept at 4°C, until removed. The amplified products were directly loaded on 2% agarose gel with 0.5 *μ*g/mL of ethidium bromide and electrophoresed in 0.5X TBE buffer for 30 minutes at 80V. The reaction pattern was photographed and analyzed for HLA-B* 27 allele. A 100 bp DNA ladder was used as a molecular size marker.

Technique was validated by retyping control samples (90) which were of 13th International Histocompatibility Working Group reference samples by PCR reverse line strip sequence specific oligonucleotide hybridization (PCR-SSOP) strips (Dynal, Invitrogen, Germany). We blindly typed these samples by in-house PCR-SSP technique and checked with HLA alleles of the same samples. Results showed 100% accuracy in newly developed technique. Each strip for HLA-B typing carried a total of 62 immobilized SSOs. Genomic DNA was amplified using HLA-B locus specific biotinylated primers and hybridized with the SSO strips. Streptavidin-horseradish peroxidase (SA-HRP) conjugates for positive color development were used with hydrogen peroxide (H_2_O_2_) and tetramethylbenzidine (TMB) as substrate. The alleles were determined using the pattern interpretation software (PMP) supplied with the kit. The antigen frequencies, odds ratio, 95% confidence interval, chi-square with Yates correction, and *P* value were estimated using SPSS software.

## 3. Results

**Figure 1:**
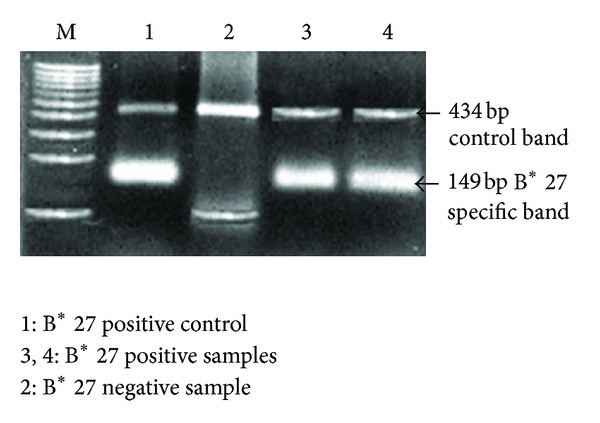
Gel photograph showing B* 27 specific band with the control bands.

The presence of a 149 bp specific band along with 434 bp control band on the 2% agarose gel (Figure 1) was indicative of HLA-B* 27 positivity. No discrepancies were seen between PCR-SSP and SSOP techniques in the 90 samples from healthy controls tested. Increased frequency of B* 27 was observed among patients when compared with normal controls (% PF 68.57 versus 4.4: O.R 46.909: 95% CI 16.993–129.49: *χ* ^2^ *Y* 135.50: *P* value 6.62*E* − 32).

In this study the age of patients ranged from 5 to 68 years with the male-to-female ratio of 4.5 : 1. When the type of arthritis was analyzed, it showed that 54% patients had polyarticular arthritis, 31% had pauciarticular arthritis, and 13% had monoarticular arthritis. Schober's test used to evaluate the restricted spine flexion (less than 5 cm) and diminished chest expansion (less than 5 cm) showed that 60% of patients had restricted spine flexion and 32% of patients had diminished chest expansion. SI joints involvement was 68%, knee joints 51%, hip joints 50%, and ankle joints 35%.

## 4. Discussion

HLA-B27 shows a remarkable association with AS and related SpA and plays a major role in disease pathogenesis. Until recently, serological MLCT and flow cytometric methods had been the conventional techniques used for HLA-B27 typing. Due to the unavailability of the specific antisera and essential use of fresh sample, these techniques are cumbersome. There are high chances of false positive and/or negative results in serology due to altered antigenic epitopes [22]. Molecular typing techniques are more specific and sensitive than traditional MLCT and flow cytometry [27]. Most molecular HLA typing methods are based on the group-specific amplification by PCR where the PCR-SSP technique is widely used to detect HLA-B* 27 [20].

In this study, we developed an in-house PCR-SSP test which amplifies all the HLA-B* 27 alleles (27:01–27:73) except B* 27:18 and B* 27:23, which have not been reported from Asian population. In our study, preparing the cocktail preparation is simple and the whole experiment can be performed in 2 hours 15 minutes. The technique developed is more cost effective than the commercial kit (present in-house PCR-SSP technique is 50 times cheaper than commercial one as well as flowcytometry technique).

The present study shows a high percentage phenotype frequency (69%) and it corroborates with many of the earlier studies reported from India [4–6, 8–15] and world [1, 7]. In conclusion, we have successfully developed a simple, convenient, and cost-effective PCR-SSP technique for HLA-B* 27 typing which is a reliable diagnostic test for AS and related SpA diagnosis. This test can now be routinely applied for HLA-B* 27 genotyping in all tissue typing laboratories.

## Figures and Tables

**Table 1:** Distribution of HLA-B27 in SpA in Indian population.

Place/population	Disease	Patients	Controls	O.R	*χ* ^2^ *Y*	*P* value	Ref. no.
		*n*+ (%PF)	*n*+ (%PF)				
(1) Pune/WI	AS	29 (72.5)	8 (9.09)	26.00	50.76	2.22*E* − 13	[8]
(2) Pune/WI	uSpA	7 (41.17)	8 (9.09)	07.00	09.50	5.38*E* − 04	[8]
(3) Mumbai/WI	SSA	41 (51.89)	1 (0.90)	117.60	66.24	9.06*E* − 17	[9]
(4) Mumbai/WI	SSA	63 (41.72)	100 (6.17)	10.88	204.65	2.41*E* − 47	[10]
(5) Chennai/SI	AS	50 (76.92)	0 (0.0)	811.26	125.77	4.78*E* − 30	[11]
(6) Chennai/SI	ReA	22 (59.45)	0 (0.0)	361.45	80.43	2.44*E* − 20	[11]
(7) Chennai/SI	uSpA	10 (40.00)	0 (0.0)	168.68	46.97	3.06*E* − 13	[11]
(8) Varanasi/NI	SSA	48 (43.63)	4 (4.0)	18.85	42.06	3.01*E* − 11	[12]
(9) Varanasi/NI	JSpA	50 (71.42)	4 (4.0)	60.00	83.29	1.49*E* − 20	[13]
(10) Varanasi/NI	SSA	151 (68.64)	4 (4.0)	52.52	112.43	7.86*E* − 27	[14]
(11) Pune/WI	SSA	253 (56.00)	7 (3.50)	35.23	157.61	1.25*E* − 36	[15]
(12) Mumbai/WI	**SpA**	**480 (68.57)**	**4 (4.40)**	**46.909**	**135.50**	**6.62*E* − 32**	**Present study**

NI: North India; WI: Western India; SI: South India; *n*+: number positive, PF: phenotype frequency, O.R: odds ratio, *χ* ^2^ *Y*: chi-square with Yates correction.
